# The first appointment with a nephrologist: Brazilian patients’ demographic and kidney function characteristics. A retrospective study

**DOI:** 10.1590/1516-3180.2021.0194.R1.13082021

**Published:** 2022-04-11

**Authors:** Farid Samaan, Danilo Euclides Fernandes, Gianna Mastroianni Kirsztajn, Ricardo Sesso

**Affiliations:** I MD, PhD. Nephrology Project Manager, Municipal Health Department of Santana de Parnaíba, Santana de Parnaíba (SP), Brazil; National Nephrology Coordinator, Grupo NotreDame Intermédica, São Paulo (SP), Brazil; Former Nephrology Coordinator, Hospital Leforte, São Paulo (SP), Brazil; and Postdoctoral Researcher, Department of Medicine, Universidade Federal de São Paulo (UNIFESP), São Paulo (SP), Brazil.; II MD. Doctoral Student, Department of Medicine, Division of Nephrology, Universidade Federal de São Paulo (UNIFESP), São Paulo (SP), Brazil.; III MD, PhD. Associate Professor of Medicine, Department of Medicine, Universidade Federal de São Paulo (UNIFESP), São Paulo (SP), Brazil.; IV MD, PhD. Full Professor of Medicine, Department of Medicine, Universidade Federal de São Paulo (UNIFESP), São Paulo (SP), Brazil.

**Keywords:** Nephrology, Primary health care, Renal insufficiency, chronic, Referral, Appointment, Primary health care, Chronic kidney disease

## Abstract

**BACKGROUND:**

The number of nephrologists has risen slowly, compared with the prevalence of chronic kidney disease (CKD) in Brazil. Data on patients referred to nephrology outpatient clinics remains scarce.

**OBJECTIVE:**

To determine the demographic and kidney function characteristics of patients at their first appointment with a nephrologist.

**DESIGN AND SETTING:**

Retrospective study conducted at three nephrology outpatient clinics (public and private services), in São Paulo, Brazil.

**METHODS:**

From December 2019 to February 2020, we collected patient data regarding demographics, kidney function parameters and comorbidities. We then analyzed data on 394 patients who met a nephrologist for their first appointment.

**RESULTS:**

The main comorbidities were hypertension (63.7%), diabetes (33.5%) and nephrolithiasis (22.3%). Regarding CKD stages, 24.1%, 9.1%, 13.7%, 15.2%, 15.2% and 2.3% of the patients were in stages 1, 2, 3a, 3b, 4 and 5, respectively. Proteinuria was absent or mild, moderate and high in 17.3%, 15.2% and 11.7%, respectively; and 16.2% had not undergone previous investigation of serum creatinine or proteinuria (55.8%). For 17.5%, referral to a nephrologist occurred late. Patients in public services were older than those in private services (59 years versus 51 years, respectively; P = 0.001), more frequently hypertensive (69.7% versus 57.5%; P = 0.01) and reached a nephrologist later (22.4% versus 12.4%; P = 0.009).

**CONCLUSION:**

Referrals to a nephrologist were not being made using any guidelines for CKD risk and many cases could have been managed within primary care. Late referral to a nephrologist happened in one-fifth of the cases and more frequently in the public service.

## INTRODUCTION

The number of nephrologists in Brazil increased by 25%, from 3,500 to 4,400 between 2008 and 2018.^1^ Within the same ten years, the number of patients on maintenance dialysis rose by 52%, from 87,044 to 133,000, approximately.^2^ Nephrologists are distributed differently across the country. While the northern region has 0.7 nephrologists per 100,000 inhabitants, the southern and southeastern regions have 6.7 nephrologists per 100,000 inhabitants.^1^ This disproportion between the numbers of nephrologists and the numbers of patients who need them is seen worldwide, even in developed countries.^3^ In the United States, between 1996 and 2012, the number of patients who started dialysis rose from 300,000 to 500,000, while the number of nephrologists decreased from 18 to only 10 per 1,000 patients.^3,4^

According to KDIGO (Kidney Disease Improving Global Outcomes), every person who presents chronic kidney disease (stages 4 and 5) and the ones who have high levels of albuminuria (albumin to creatinine ratio > 300 mg/g) should be referred to a nephrologist.^5^ The Brazilian Ministry of Health also recommends that patients who are in stages 4 or 5 of chronic kidney disease (CKD) should be followed up by a specialist.^6^

Delay in reaching a nephrologist is associated with unfavorable outcomes and higher healthcare expenditure.^7-11^ In Brazil, late referral to a nephrologist was first demonstrated in 1995.^12^ In that study, about 60% of the patients who started dialysis had not been followed up on an outpatient basis by a nephrologist.

On the other hand, some studies have suggested that many patients who are referred to a nephrologist can be easily followed up within primary care.^13,14^ Bahiense-Oliveira et al. showed that 52% of the patients assisted by a nephrologist did not need to be assessed or treated by this specialty at their first appointment.^13^ Another study showed that 35.7% of the patients assessed by nephrologists had stages 1 and 2 of CKD and only a few of them (26%) presented higher levels of proteinuria or albuminuria, meaning that many patients could have continued to be cared for within primary care.^14^

## OBJECTIVE

Because of the need for accurate medical referral to nephrologists and the lack of these specialists, the aim of this study was to describe the characteristics (sociodemographic and CKD stages) of patients who were assessed by nephrologists at their first appointment, in both public and private services.

## METHODS

### Study design and participants

This was a multicenter retrospective study based on medical records. We included three outpatient clinics in the metropolitan area of São Paulo: two clinics affiliated with private health insurance services and one public clinic within the Brazilian National Health System (Sistema Único de Saúde, SUS). We analyzed information on first appointments with a nephrologist that took place between December 2019 and February 2020, among patients who were ≥ 18 years old. We excluded those who had undergone kidney transplantation or who were on kidney replacement therapy. In the Brazilian public service, patients can only reach specialists through a medical referral from primary care or from other specialists. In private services, patients can reach specialists either through referrals or through their own initiative.

The protocol for this study was approved by the Ethics Committee of Universidade Federal de São Paulo on June 5, 2020 (CAAE 31053420.9.1001.5505).

### Definitions and parameters of interest

Basic characteristics and clinical information relating to diagnoses of hypertension, diabetes mellitus (DM), urinary lithiasis, recurrent urinary tract infection, polycystic kidney disease and glomerulonephritis were obtained from the patients’ charts. We defined hypertension as the use of anti-hypertensive drugs or the presence of this diagnosis in the patient’s chart. DM was defined from use of oral antidiabetic drugs or insulin therapies or the presence of this diagnosis in the patient’s chart.

Laboratory assessments included serum creatinine and proteinuria. We used serum creatinine, age, and gender to determine the estimated glomerular filtration rate (eGFR), in accordance with the Chronic Kidney Disease Epidemiology Collaboration (CKD-EPI) equation.^15^ The racial factor was not included in the eGFR calculation because of the multiethnic composition of the Brazilian population and because of a previous study that demonstrated that this adjustment did not contribute to greater accuracy in this population.^16^ CKD was defined as eGFR < 60 ml/min/1.73 m^2^ or the presence of biomarkers for renal dysfunction, such as proteinuria, dysmorphic hematuria or abnormal kidney ultrasound. We classified CKD into five stages in accordance with the current guidelines: stage 1 (eGFR ≥ 90 ml/min/1.73 m^2^ and any renal dysfunction biomarker); stage 2 (eGFR of 60-89 ml/min/1.73 m^2^ and any renal dysfunction biomarker); stage 3a (eGFR of 45-59 ml/min/1.73 m^2^), stage 3b (eGFR of 30-44 ml/min/1.73 m^2^); stage 4 (eGFR of 15-29 ml/min/1.73 m^2^); and stage 5 (eGFR < 15 ml/min/1.73 m^2^). We considered stages 3b, 4 and 5 to be advanced CKD.^5^

We used the following methods to determine the levels of proteinuria: urinalysis (dipstick); random urinary albumin-to-creatinine ratio (ACR); random urinary protein-to-creatine ratio (PCR); 24-hour albuminuria; and 24-hour proteinuria. We stratified the patients into three categories according to their level of proteinuria: absent or mild (urinalysis negative or 1+, ACR < 30 mg/g, PCR < 150 mg/g, 24-hour albuminuria < 30 mg or 24-hour proteinuria < 150 mg); moderate (1+ or 2+ on urinalysis, ACR of 30-300 mg/g, PCR of 150-500 mg/g, 24-hour albuminuria of 30-300 mg and 24-hour proteinuria of 150-1000 mg); and high (3+ on urinalysis, ACR > 300 mg/g, PCR > z500 mg/g, 24-hour albuminuria > 300 mg and 24-hour proteinuria > 1000 mg).^5^

Among the reasons for referring patients to a nephrologist, we considered the following: hypertension, diabetes, nephrolithiasis, recurrent urinary tract infection, hematuria (red blood cells above the laboratory reference levels) and acute kidney injury (serum creatinine > 0.3 mg/dl, in comparison with the baseline serum creatinine, investigated within the preceding three months before data collection).^17^ We collected data on the specialties from which patients were referred to a nephrologist (internal medicine, endocrinology, cardiology or urology). Also, we registered whether patients reached a nephrologist by themselves, with no medical referral. Late referral was defined as referral in stages 4 or 5.^7-11^

Patients for whom serum creatinine and proteinuria information was available were classified into CKD risk groups: low risk, moderate risk, high risk and very high risk.^18^

#### Sampling and statistical analyses

We calculated the sample size based on the following equation:^19^ N = n*X / (X + n – 1); in which X = Z_(α/2)_^2^ *p*(1-p) / error^2^. “Z_(α/2)_” is the critical value for a normal distribution when α/2 (confidence interval = 95%, α = 0.05 and critical value of 1.96), “p” represents the proportion of the referred patients in the sample, “error” is the estimated margin around “p”, and “n” means the size of the population. We estimated that 40% of the patients were correctly referred to a nephrologist, in conformity with previous research.^12,13^ We considered that the size of the population was 100,000 inhabitants. We set error and confidence intervals of 8% and 95%, respectively. In this manner, we determined that a minimum of 144 medical records from public and private services would need to be analyzed.

We used the statistical package SPSS, version 18.0 (SPSS Inc., Chicago, Illinois, United States). We described the frequencies of the categorical variables. Age showed non-normal distribution and so we presented data on its median and interquartile range (IQR). We used χ^2^ or Fisher exact tests to compare the frequencies, as appropriate. Also, we used the Mann-Whitney test to compare non-normal continuous variables. We set the significance level for P-values at < 0.05.

## RESULTS

The demographic and clinical characteristics (renal data and comorbidities) of our sample are shown in Table 1. The median age was 55 years old (IQR, 42-67); 51.5% of the patients were male; and the most frequent comorbidities were hypertension (63.7%), DM (33.5%), nephrolithiasis (22.3%) and recurrent urinary infection (8.6%). The distribution of the patients regarding CKD grading was 24.1% in stage 1, 9.1% in stage 2, 13.7% in stage 3a, 15.2% in stage 3b, 15.2% in stage 4 and 2.3% in stage 5.

**Table 1.** Characteristics of the patients and comparison between public and private healthcare services

	All (n = 394)	Public (n = 201)	Private (n = 193)	P-value*
**Age, years (range)**	55 (42-67)	59 (47-69)	51 (38-64)	0.001
**Male, n (%)**	203 (51.5)	97 (48.3)	106 (54.9)	0.18
**Reasons for referring to a nephrologist/comorbidities, n (%)**				
Hypertension	251 (63.7)	140 (69.7)	111 (57.5)	0.01
Diabetes mellitus	132 (33.5)	74 (36.8)	58 (30.1)	0.15
Nephrolithiasis	88 (22.3)	41 (20.4)	47 (24.4)	0.35
Recurrent urinary tract infection	34 (8.6)	19 (9.5)	15 (7.8)	0.55
Polycystic kidney disease	15 (3.8)	8 (4.0)	7 (3.6)	0.85
Glomerulonephritis	4 (1.0)	1 (0.5)	3 (1.6)	0.29
Acute kidney injury	14 (3.6)	8 (4.0)	6 (3.1)	0.64
Hematuria	8 (2.0)	4 (2.0)	4 (2.1)	0.95
Other	59 (15.0)	18 (9.0)	41 (21.2)	0.001
**CKD stage, n (%)**				
1	95 (24.1)	41 (20.4)	54 (28.0)	0.07
2	36 (9.1)	23 (11.4)	13 (6.7)	0.11
3a	54 (13.7)	31 (15.4)	23 (11.9)	0.31
3b	60 (15.2)	39 (19.4)	21 (10.9)	0.02
4	60 (15.2)	43 (21.4)	17 (8.8)	< 0.001
5	9 (2.3)	2 (1.0)	7 (3.6)	0.08
**Proteinuria stratification, n (%)**				
Absent or mild	68 (17.3)	28 (13.9)	40 (20.7)	0.74
Moderate	60 (15.2)	40 (19.9)	20 (10.4)	0.008
High	46 (11.7)	30 (14.9)	16 (8.3)	0.58
**Referral with no serum creatinine result, n (%)**	64 (16.2)	22 (10.9)	42 (21.8)	0.004
**Referral with no proteinuria result, n (%)**	220 (55.8)	103 (51.2)	117 (60.6)	0.06
**No CKD, n (%)**	16 (4.1)	0 (0.0)	16 (8.3)	< 0.001
**Advanced CKD, n (%)** **	129 (32.7)	84 (41.8)	45 (23.3)	< 0.001
**Late referral, n (%)** ***	69 (17.5)	45 (22.4)	24 (12.4)	0.009
**Referred from, n (%)**				
No referral	72 (18.3)	0 (0.0)	72 (37.3)	< 0.001
General practitioner (internist)	228 (57.9)	187 (93.0)	41 (21.2)	< 0.001
Endocrinologist	28 (7.1)	3 (1.5)	25 (13.0)	< 0.001
Urologist	11 (2.8)	0 (0.0)	11 (5.7)	0.002
Other	55 (14.0)	11 (5.4)	44 (22.8)	< 0.001
**Discharge from the nephrologist**	39 (9.9)	7 (3.5)	32 (16.5)	< 0.001

* Public versus private;

** Chronic kidney disease (CKD) stages 3b, 4 and 5;

*** CKD stages 4 and 5.

Proteinuria was observed to be mild or absent in 17.3% of the patients, while 15.2% presented moderate and 11.7% presented high levels of proteinuria. The percentages of patients referred to a nephrologist with no information on serum creatinine and proteinuria were 16.2% and 55.8%, respectively. Among patients with hypertension and without DM, the referral rates for those with results from laboratory tests on serum creatinine and proteinuria were 87% and 48%, respectively. Among those with DM, the corresponding referral rates reached 91% and 56%, respectively.

Late referral (stages 4 and 5) was found in 17.5% of the participants. Most of the patients (57.9%) were referred to a nephrologist by a general practitioner. About 10% of the patients were discharged by the nephrologist after the first appointment.

Compared with the private insurance patients, the individuals seen in public outpatient clinics were older (59 [IQR, 47-69] versus 51 [IQR, 38-64] years old; P = 0.001), more commonly hypertensive (69.7% versus 57.5%; P = 0.01) and were referred later to a nephrologist (22.4% versus 12.4%; P = 0.009). In the public service, patients were more frequently referred to a nephrologist without serum creatinine results (10.9% versus 21.8%, respectively; P = 0.004) and without proteinuria tests (51.2% versus 60.6%; respectively, P = 0.06). The type of doctor who most frequently referred patients to a nephrologist was the general practitioner (93%) in the public service; while in the private service patients reached a nephrologist predominantly without any referral (37.3%) followed by referral from a general practitioner (21.2%).

According to the risk map for CKD,^19^ 19.9% of the patients were at low risk, 21% at moderate risk, 24% at high risk and 34.8% at very high risk (Table 2). Compared with patients seen at private outpatient clinics, those seen within the public healthcare system presented lower probability of being at low risk of CKD (11.2% versus 32.4%, respectively; P < 0.001) and higher risk of CKD (69.4% versus 44.1%; respectively, P = 0.001) (Table 3).

**Table 2.** Patients’ distribution* according to the risk map for chronic kidney disease^19^

Chronic kidney disease stages	Estimated glomerular filtration rate** (ml/min/1.73 m^2^)	Proteinuria stratification		
		Absent or mild n (%)	Moderate n (%)	High n (%)
1	> 90	26 (15.7)^a^	19 (11.4)^b^	9 (5.4)^c^
2	60-89	7 (4.2)^a^	10 (6.0)^b^	9 (5.4)^c^
3a	45-59	6 (3.6)^b^	12 (7.2)^b^	7 (4.2)^d^
3b	30-44	10 (6.0)^c^	11 (6.6)^d^	13 (7.8)^d^
4	15-29	12 (7.2)^d^	8 (4.8)^d^	4 (2.4)^d^
5	< 15	0 (0.0)^d^	0 (0.0)^d^	3 (1.8)^d^

* 166 patients for whom data on serum creatinine and proteinuria were available. Risk map according to the Kidney Disease Outcomes Quality Initiative (KDOQI);

** CKD-EPI equation;

a low risk;

b moderate risk;

c high risk;

d very high risk.

**Table 3.** Comparison between public and private health insurance patients regarding the risk of chronic kidney disease^19^

Risk stratum	Public (n = 98)	Private (n = 68)	P
Low	11 (11.2)	22 (32.4)	< 0.001
Moderate	19 (19.4)	16 (23.4)	0.52
High or very high	68 (69.4)	30 (44.1)	0.001

## DISCUSSION

The Brazilian guidelines regarding CKD define that the risk stratification should be conducted within primary care through assessing serum creatinine and proteinuria levels.^6^ The Brazilian guidelines for hypertension and DM also include serum creatinine and proteinuria tests performed annually, as a minimum.^20^

This study showed that for one patient in six, no information on serum creatinine was available at the time of the first appointment with a nephrologist. Additionally, more than half of the patients were not investigated regarding urinary protein levels. Failure in screening for CKD has also been observed in other regions in which the rates of serum creatinine monitoring (32.5% to 73.5%) and proteinuria assessment (2.5% to 40%) were low.^21-23^

Considering the impact of aging on the decline in renal function,^24^ 23 patients (5.9% of the sample) may not necessarily have needed to be referred to a nephrologist (patients aged > 75 years; eGFR < 60; and proteinuria assessment not performed or absent). Nonetheless, most of them were referred without any assaying of proteinuria (20 patients).

The prevalences of hypertension and DM in our sample were 63.7% and 33.5%, respectively. According to a survey by the Brazilian Nephrology Society,^2^ the most common causes of CKD stage 5 are hypertension and DM. Indeed, these diseases can be identified and prevented within primary care.^25-28^ Proteinuria plays an important role in accelerating the progression rate of CKD, but its assessment was neglected among 52% of hypertensive and 44% of diabetic patients. Although testing of proteinuria levels is important, this was not usually performed.

Compared with the patients seen via the private healthcare service, the public service patients who reached a nephrologist showed higher rates of advanced CKD (stages 3b, 4, and 5), but serum creatinine and proteinuria were more frequently assessed before the referral. Because of the scarcity of nephrology appointments within the public service, those individuals may reach the specialist later in time, and with advanced stages of CKD. This may also happen because patients with hypertension or DM are highly adherent to their treatment within primary care. Public service patients had better chances of undergoing CKD screening, probably due to the higher degree of control and requirements for setting up appointments with specialists. Doctors who work for SUS need to provide written justification in advance, to explaining why the patient should be referred to a specialist.

The main type of physician responsible for referring patients to a nephrologist within the public service was the general practitioner, while in private healthcare services there was a broader range of sources such as self-referral, general practitioners and other specialists, thus suggesting that the private healthcare service is highly compartmentalized.

About 25% of the patients referred to a nephrologist did not belong to the major risk groups for CKD (hypertension, DM, elderly people, polycystic kidney disease and glomerulonephritis). This percentage was greater in the public service than in private services (31.1% versus 19.9%, respectively; P = 0.01). Patients at low risk of CKD were more commonly seen in the private healthcare services. These results suggest that there are higher rates of unnecessary appointments with specialists within private services, which can be explained by the convenience of reaching a specialist when the patient can afford it.

Some limitations of this study should be mentioned. First, this was a retrospective study, and some of the patients could not take their laboratory results to their first appointment with the nephrologist, which may have prevented nephrologists from registering patients’ lab results in the charts. Second, the limited number of centers included in this study prevented us from generalizing our results to other public or private Brazilian healthcare services. Third, the data available in relation to diabetes and hypertension (diagnosis and pharmacological treatment) may have provided an underestimate of their prevalences, especially because patients with diabetes and hypertension are oligosymptomatic at the beginning of their natural history. However, the result that we have presented through our sampling may work as a comparison for other, future research, in order to educate both patients and healthcare professionals about the early stages of CKD.

## CONCLUSIONS

There are opportunities to improve the stratification of the risk of chronic kidney disease (CKD) in both public and private healthcare services. Proteinuria plays an important role in predicting CKD and seems to have been ignored in many patients who are at high risk of CKD, such as hypertensives and diabetics. Late referral to a nephrologist and unnecessary appointments with this specialist are common in public and private services, respectively. Further research aimed at monitoring healthcare quality in the early stages of CKD may improve the way in which physicians refer their patients to a nephrologist.
